# UBR1 is a prognostic biomarker and therapeutic target associated with immune cell infiltration in gastric cancer

**DOI:** 10.18632/aging.206079

**Published:** 2024-08-23

**Authors:** Weiwei Yuan, Jianye Han, Chen Chen, Yue Qiu, Yuanmin Xu, Yang Huang, Zhangming Chen, Aman Xu, Minzhi Sun

**Affiliations:** 1 Department of Thyroid Surgery, Baoshan Hospital Affiliated to Shanghai University of Traditional Chinese Medicine, Shanghai 201999, China; 2 Department of General Surgery, The First Affiliated Hospital of Anhui Medical University, Anhui Public Health Clinical Center, Hefei 230012, China; 3 Department of General Surgery, The First Affiliated Hospital of Anhui Medical University, Hefei 230022, China

**Keywords:** UBR1, stomach adenocarcinoma (STAD), immunotherapy, prognostic biomarker, immune cell infiltration

## Abstract

Background: Ubiquitination is a targeted protein modification process mediated by intracellular molecules. UBR1 encodes a protein that binds to unstable N-terminal residues of substrate proteins and contributes to the formation of substrate-linked polyubiquitin chains. However, the function and cellular pathways of UBR1 in tumors have received inadequate attention. This study aimed to investigate the potential of UBR1 as a prognostic biomarker and immunotherapy target for stomach adenocarcinoma (STAD) as well as its biological function and molecular mechanism in relation to the disease.

Methods: Differential expression and pan-cancer gene set enrichment analysis (GSEA) were conducted using The Cancer Genome Atlas (TCGA), Gene Expression Omnibus (GEO), and Genotype-Tissue Expression (GTEx) datasets. The Human Protein Atlas (HPA) database was utilized to identify UBR1-enriched pathways in AGS cells and to compare immunohistochemical differences between cancerous and adjacent non-cancerous tissues in gastric cancer. Quantitative Polymerase Chain Reaction (QPCR) and Western blot (WB) analyses were employed to validate these findings in both cancerous and adjacent non-cancerous tissues of gastric cancer. UBR1 expression in GES-1 and four gastric cancer cell lines was assessed using QPCR and WB. Kaplan-Meier curves, univariate and multivariate Cox regression analyses, and receiver operating characteristic (ROC) curve analyses were performed to evaluate the prognostic and diagnostic roles of UBR1. Additionally, the correlation between UBR1 expression and clinical parameters was analyzed using TCGA and GEO databases. UBR1 mutation data were obtained from the cBioPortal database. The mutation landscape, mutation-associated genes, protein structure, tumor mutation burden (TMB), and microsatellite instability (MSI) correlations were analyzed and illustrated. The biological functions of UBR1 were investigated using Gene Ontology (GO) and Kyoto Encyclopedia of Genes and Genomes (KEGG) enrichment analyses. The correlation between UBR1 and immune infiltration was assessed using TIMER and EPIC computational methods. Protein expression levels of UBR1 in gastric cancer cell lines were determined by immunohistochemistry (IHC) and WB analysis. Quantitative real-time PCR (qRT-PCR) was employed to analyze mRNA expression. Immunoprecipitation (IP) assays were conducted to detect protein-protein interactions between UBR1 and PDL1, while cellular immunofluorescence was used to observe the co-localization of these proteins. Cell proliferation was evaluated using CCK8 and colony formation assays. Cell migration was assessed using Transwell and wound healing assays. Finally, apoptosis was analyzed using flow cytometry, and WB was used to detect changes in apoptotic proteins and NF-κB P65 pathway proteins.

Results: UBR1 was upregulated in 28 cancer types, including STAD, and its overexpression was validated in gastric cancer cell lines and tissues. UBR1 expression was associated with advanced pathological characteristics. High UBR1 expression was linked to poor prognostic outcomes, including overall survival (OS), progression-free interval (PFI), disease-specific survival (DSS), as well as responses to surgery, chemotherapy, and HER2 expression. UBR1 expression showed significant correlations with clinical parameters such as age, gender, TNM stage, pathological stage, tumor resection, and anti-reflux therapy. Amplifications and deletions were the most frequent genetic alterations associated with UBR1. According to KEGG and GSEA analyses, UBR1 was significantly associated with several cancer pathways, oxidative phosphorylation, and the TNF-NFκB pathway. UBR1 also exhibited a significant correlation with immune cell infiltration and immunotherapy, including a direct interaction with PDL1. Knockdown of UBR1 inhibited the proliferation, migration, and invasion of STAD cells and promoted apoptosis.

Conclusions: UBR1 is overexpressed in STAD, promoting its progression and positively correlating with immune cell infiltration and immunotherapeutic responses. Therefore, UBR1 could be a promising biomarker for the prognosis and immunotherapy of STAD.

## INTRODUCTION

Gastric cancer (GC) is a prevalent form of cancer globally, ranking fifth in incidence and fourth in cancer-related deaths. patients with advanced gastric cancer have a median survival period of less than 12 months [1]. Several factors, including geography, infection with Helicobacter pylori (HP) and Epstein-Barr virus (EBV), diet, and genetic mutations, influence the incidence of GC [2, 3]. Currently, surgery is the primary treatment for early- to mid-stage GC. For locally advanced or distant metastatic GC, surgery is combined with chemotherapy, radiotherapy, immunotherapy, and molecular targeted therapy [4–6]. Despite the rapid and potent effects of chemotherapy and targeted drugs, patients with GC frequently develop drug resistance over time. Although immunotherapy may have a delayed onset, it can yield substantial benefits for patients [7, 8], with some individuals experiencing long-term survival or even complete remission. Therefore, the identification of immunotherapeutic prognostic markers for GC is crucial for enhancing patient survival and identifying novel drug targets.

Ubiquitination is a crucial biochemical process in which specific enzymes catalyze the covalent attachment of ubiquitin to target protein molecules, thereby exerting extensive biological functions. In this process, ubiquitin molecules are activated and attached to target proteins, altering their function or marking them for degradation. Ubiquitination typically involves the synergistic action of three key enzymes. First, the E1 ubiquitin-activating enzyme activates ubiquitin molecules through ATP hydrolysis. Second, the E2 ubiquitin-conjugating enzyme transfers the activated ubiquitin to its own active site. Finally, the E3 ubiquitin ligase recognizes specific target proteins and transfers ubiquitin from the E2 enzyme to the target protein [9]. E3 proteins play a critical role in this process because they determine the specificity and selectivity of the ubiquitination reaction. E3 proteins regulate various cellular processes, including but not limited to protein degradation, cell signaling, autophagy, apoptosis, transcriptional activation, and translation [10]. UBR1, the first ubiquitinylating protein discovered, is a single-subunit E3 ligase that selectively binds to proteins with specific N-terminal residues [11]. Recent studies have combined protein chemical synthesis and cryo-electron microscopy to investigate the dynamic process of ubiquitin E3 ligase UBR1 recognizing and modifying substrates using K48 chain-type polyubiquitination. This provides further insights into substrate ubiquitination initiation and ubiquitin chain extension [12]. The human genome encodes over 600 E3 enzymes, some of which are crucial targets for various drug therapies. UBR1 is linked to various human diseases, including steatosis [13, 14], pancreatic dysfunction, malformation, Johnson-Blizzard syndrome (JBS) [15] and cancer. However, the function and regulatory mechanisms of UBR1 in GC have not yet been documented.

Increasing evidence suggests that the onset, development, and recurrence of many cancers are associated with T-cell dysfunction and immunosuppression mediated by immunological checkpoint molecules [7, 16]. Immunotherapy induces tumor destruction by activating host immune responses. Immune checkpoint-related biomarkers, including programmed death receptor 1 (PD-1), programmed cell death 1 ligand 1 (PD-L1), and cytotoxic T lymphocyte-associated antigen 4 (CTLA-4), are involved in this process [17]. Current cancer immunotherapy research focuses on immune checkpoint inhibitor (ICI) combination therapy [18, 19]. Although various immunotherapies have been used in combination with inhibitors for several types of cancers, patient benefits remain limited. Therefore, there is an urgent need to identify new immunological markers. We investigated the effect of UBR1 on the immunological response to GC.

This study aimed to investigate the role of UBR1 in GC diagnosis and prognosis using bioinformatics analyses. It also evaluated UBR1 expression levels in GC at both the tissue and cellular levels and analyzed the role of UBR1 in GC development at the cellular level. The results revealed that UBR1 is a potential biomarker for gastric cancer prognosis and immunotherapy, as it positively correlates with immune cell infiltration and immunotherapeutic response and can directly affect PDL1 expression.

## MATERIALS AND METHODS

### Data source

Transcriptomic and clinical data were acquired from The Cancer Genome Atlas (TCGA) database (https://www.cancer.gov/tcga/) and normal human expression data from the GTEx database (https://www.genome.gov/), using Perl software and “limma” R package for processing and merging. UBR1 expression distribution in STAD and normal tissues from the TCGA and GTEx databases were comparatively analyzed using the GEPIA database. GEO datasets (https://www.ncbi.nlm.nih.gov/geo/) (GSE54129, GSE66229, and GSE118916) were used to validate UBR1 expression levels in gastric cancer and paraneoplasia. Pathology-specific changes in UBR1 expression were compared using the HPA database to predict pathways enriched by UBR1 in AGS gastric cancer cells. Survival analysis was performed using the Kaplan-Meier method and validated using the GEO datasets (GSE15459, GSE51105, and GSE62254). UBR1 mutations and copy number variants were analyzed using the cBioPortal database. We used TIMER and EPIC databases to investigate the correlation between UBR1 and immune cells and evaluated the predictive capability of UBR1 for immunotherapy efficacy using iMvigor78820 (http://research-pub.gene.com/IMvigor210CoreBiologies). In addition, the relationship between UBR1 expression and clinical parameters (including gender, TMN stage, and molecular subtype) was investigated using TCGA and GEO databases. Patients without complete clinical information or overall survival (OS) data were excluded from the analysis.

### Clinical tissue samples

Gastric cancer and corresponding normal paracancerous mucosal tissues were collected from 30 patients admitted to the Department of Gastroenterology Surgery at the First Affiliated Hospital of Anhui Medical University between May 2022 and 2023. The extracted protein and RNA were stored at −80°C. The patients did not receive neoadjuvant chemotherapy or immunotherapy prior to surgery and had no history of immune-related illnesses. The experimental design adhered to the requirements of the Ethics Committee of the First Affiliated Hospital of Anhui Medical University. Informed consent was obtained from all the patients or their authorized relatives.

### Immunohistochemistry (IHC)

All preoperative biopsies of the patients were pathologically diagnosed as gastric adenocarcinoma. Fresh GC and paraneoplastic tissues were fixed in 10% neutral formalin, sequentially dehydrated, embedded, blocked, sectioned, dried, and stored at room temperature. Subsequently, dewaxing and hydration procedures were performed, followed by high-pressure repair of the antigenic determinant clusters. Endogenous peroxidase blockers were added, and the cells were incubated for 15 minutes. An anti-phosphorylated UBR1 primary antibody (1:150 dilution) was added dropwise, and the mixture was incubated overnight in a wet box at 4°C. The secondary antibody (1:400 dilution) was added dropwise, and the mixture was incubated at 37°C for 20 minutes. The chromogenic agent, diaminobenzidine (DAB), was added dropwise, and the resulting color development was observed under a microscope. The sample was subsequently re-stained with hematoxylin solution for 1 minute. The dehydration process was performed sequentially using gradient alcohol, followed by sealing the films with neutral resin drops and drying them for photography. UBR1 expression levels were evaluated using histochemical scoring (H score).

### Cell immunofluorescence

Place coverslips in 24-well or 12-well plates and seed cells, culturing them until the cell density reaches 50–70%. Discard the medium and wash once with PBS. Add cold 4% paraformaldehyde to each well (400 μl for 12-well plates, 200 μl for 24-well plates) and fix for 30 minutes. Wash three times with PBS, gently shaking each time. Add 0.5% Triton X-100 (diluted in PBS) to each well for 20 minutes to permeabilize the cells, then wash three times with PBS. Add blocking solution (0.03 g BSA/300 μl for 12-well plates, 0.015 g BSA/150 μl for 24-well plates, diluted in TBXT) or goat serum for 1 hour, then wash once with PBS. Add 25 μl of primary antibody (diluted as per the instructions) to the coverslip, placing the cell side down on the antibody droplet. Place the coverslips in a humidified chamber with damp filter paper and incubate overnight at 4°C, protected from light. The next morning, return the coverslips to the 24-well plate, cell side up, and wash three times with PBS. Add 150 μl of secondary antibody (diluted as per the instructions) to each well, incubating in the dark at room temperature for 1.5 hours. Wash three times with PBS. Finally, add 30 μl of anti-fade mounting medium with DAPI to a slide, place the coverslip cell side down on the mounting medium, seal the edges with nail polish, allow to dry, and then take images.

### Genetic alterations

The genetics of UBR1 alterations were queried using the cBioPortal (https://www.cbioportal.org). This study analyzed the mutation frequency, type, location, and amino acid changes in UBR1, as well as trends in mRNA expression relative to gene copy number variability (CNA).

### Analysis of survival and independent prognostic factors

Transcriptomic and clinical data of patients with STAD were obtained from the TCGA database. Subsequently, patients were categorized into high- and low-risk groups based on UBR1 expression in gastric cancer, and survival curves for overall survival (OS), progression-free interval (PFI), and disease-specific survival (DSS) were generated. The Kaplan-Meier plotter (https://kmplot.com/analysis/) was used to validate and plot UBR1 survival curves in the GEO (GSE15459, GSE51105, and GSE62254) datasets and to assess their clinical significance.

Transcriptional and clinical data of patients with STAD were downloaded from the TCGA website. Subsequently, the patients were categorized into high- and low-risk groups based on the median UBR1 expression levels. Univariate Cox regression analysis was used to identify clinical parameters and UBR1 expression associated with the prognosis of patients with STAD. Multivariate Cox analysis was conducted to determine independent prognostic factors with a significance level of *P* < 0.05.

### Gene set enrichment analysis (GSEA)

The differentially expressed gene set of UBR1 was identified and analyzed for gene ontology (GO) function and the Kyoto Encyclopedia of Genes and Genomes (KEGG) pathway using the clusterProfiler R package. Initially, the correlation coefficients between UBR1 and other genes were calculated. Subsequently, GSEA was performed using gene expression matrices ranked based on these correlation coefficients. Statistically significant enrichment results were those with a *P*-value < 0.05 and a false discovery rate (FDR) < 0.25.

### Immune infiltration

The tumor microenvironment, specifically the immune-to-stromal cell ratio, significantly affects prognosis. The depth of non-tumor cell infiltration can be predicted by analyzing the immune, stromal, and ESTIMATE scores. We used the Tumor Immunity Estimation Resource 2.0 (TIMER2.0; http://timer.cistrome.org/) database to determine the immune cell infiltration of UBR1 in tumors and evaluate its correlation with markers of immune cell subsets, including CD8+ T cells, CD4+ T cells, B cells, monocytes, tumor-associated macrophages (TAM), neutrophils, NK cells, DCs, Tregs, and other immune cell subsets.

### Cell culture and siRNAs

Several human gastric cancer cell lines (GES-1, MGC803, MKN45, AGS, and HGC27) were cultured in a medium containing 90% RPMI-1640 (Gibco, USA) and 10% fetal bovine serum (Wisent, Canada). Cells were transfected with 100 nM scrambled siRNA at 50% confluence in six-well plates using Lipofectamine 2000 as the transfection reagent. Transfected cells were collected to assess knockdown efficiency by immunoblotting and RT-qPCR using a UBR1 antibody (dilution 1:1000; 26069-1-AP, Proteintech Group, Wuhan, China) for validation. Total RNA was extracted from the transfected cells using TRIzol reagent (Invitrogen, USA) according to the manufacturer’s instructions. The extracted RNA was reverse transcribed into cDNA using a PrimeScript RT reagent kit (Takara, Japan). The cDNA was then used as a template for qPCR amplification. The qPCR was performed using SYBR Green Master Mix (Applied Biosystems, USA) on a QuantStudio 3 Real-Time PCR System (Applied Biosystems, USA). The following siRNA sequences were used: siUBR1#1, 5′-UCAAAU AGCAUCAAGGAAATT-3′; siUBR1#2, 5′-GGAUGA AUAUGGAGAAACATT-3′; The primers used were: UBR1 forward sequence: 5′-TGCCGACTACAAGCGA ATTACTG-3′ and reverse sequence: 5′-CTGCTTGTC CAGATGACTTCGG-3′.

### Immunoprecipitation (IP)

MGC803 cells were washed twice with pre-cooled PBS, and 1 ml of cell lysate containing lysis buffer, protease inhibitors, and phosphatase inhibitors was added. The cells were lysed on ice for 30 minutes, scraped, and centrifuged. A 50 μL supernatant was collected as the input sample, while the remaining supernatant was transferred to a new 2 ml EP tube for the IP and IgG samples. UBR1 and IgG antibodies were added to the respective samples at 4°C. Following overnight incubation, protein A/G beads were added, and the supernatant was discarded. IP lysate and protein loading buffer were added, and the mixture was heated at 100°C for 10 minutes. The samples were subsequently analyzed by Western blotting.

### Apoptosis

Seed the required gastric cancer cell lines in six-well plates. Observe under a microscope the next day to ensure that the cell confluence reaches 80–90% and that the cells are in the logarithmic growth phase. Digest the cells into single cells using trypsin without EDTA, then collect them into a 15 ml centrifuge tube and centrifuge at 1000 rpm for 3 minutes. Resuspend the cells in cold PBS, and repeat the 1000 rpm centrifugation for 3 minutes twice, removing the supernatant each time. Take 1–5 × 10^5^ cells, centrifuge to remove the supernatant, and wash once with serum-containing medium. Resuspend the cells in 500 μl of Binding Buffer. Add 5 μl of Annexin V and 5 μl of 7-AAD, incubate at room temperature in the dark for 15 minutes, and then proceed with flow cytometry analysis.

### Cell proliferation, migration, colony formation, and wound healing assays

The cells were seeded at a density of 3000 cells/well in 96-well plates. Cell viability was determined using the Cell Counting Kit-8 (CCK-8), a cell proliferation and activity assay kit. The effect of siUBR1 on cell invasion capacity was measured using Transwell chambers. The matrix gel was applied onto the filter membrane of the chambers, followed by seeding cells at a density of 2 × 10^4^ in a low serum medium containing 1% FBS (200 μl) before being transferred to the chambers. Transwell chambers were placed in 24-well plates, and 500 μL of complete medium was added. Following a 24-hour incubation period, the cell chambers were removed, fixed in paraformaldehyde, and stained with crystal violet. The migrated cells were observed microscopically.

For testing purposes, 200 cells/well were seeded in 6-well plates with a complete medium containing 10% FBS. The culture medium was changed every three days, and the colonies were removed after two weeks. Finally, colonies were fixed with polymethanol for 20 minutes, stained with crystal violet for 30 minutes, and counted using ImageJ software.

The cells were spread across a 6-well plate and scored in a cell monolayer using a 200 μL pipette tip. The scored wells were washed twice with PBS and supplemented with a low-serum medium containing 5% FBS. Microscopic images were captured at 0 and 24 hours. Relative migration rates were calculated using ImageJ software.

### Statistical analysis

In public databases, we assessed the disparities in molecular expression between tumor and normal tissues using the Wilcoxon rank-sum test. To illustrate prognostic differences, Kaplan-Meier survival curves were employed for groups with high versus low UBR1 expression, and survival rates were compared using log-rank tests. Using Spearman’s method, we analyzed the correlation between UBR1 expression and immune cell infiltration, with further evaluation of differences in immune infiltration among these groups via the Wilcoxon rank-sum test. The variation in UBR1 expression across different cancer types was also examined using the same statistical approach. All bioinformatics analyses were conducted with R software (version 4.1.2). To ensure reliability, experimental procedures were replicated at least three times. Western blot results were quantified using ImageJ software, and inter-group differences were analyzed using the *t*-test in GraphPad Prism 8.02. We set a significance threshold of *p* < 0.05 for all statistical analyses, and results were displayed in bar graphs showing the mean ± standard deviation (SD) from the three experiments.

## RESULTS

### Expression profile and GSEA analysis of UBR1 in human cancer

**Figure 1:**
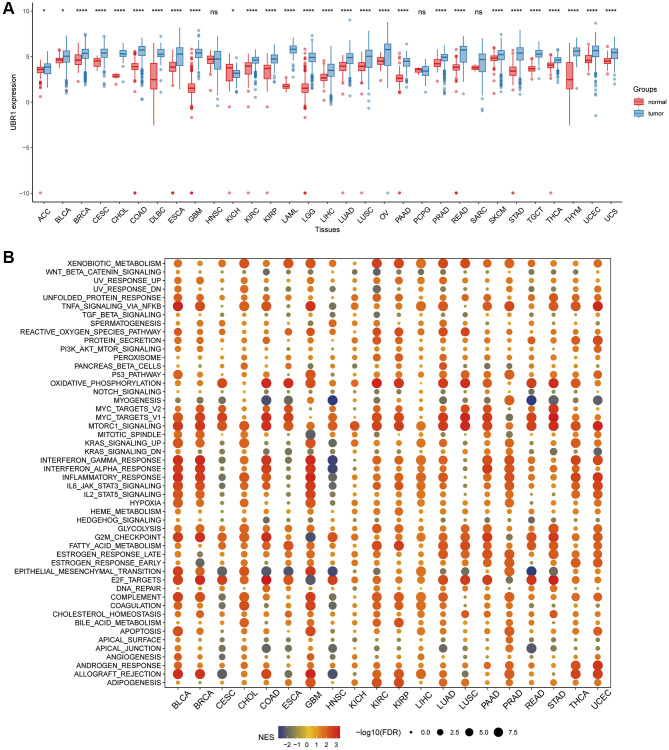
**Analysis of UBR1 expression profile and GSEA in human cancers.** (**A**) A comprehensive pan-cancer analysis was performed to evaluate UBR1 expression across various cancer and normal tissues using combined data from the TCGA and GTEX databases. Statistical significance is indicated as follows: ^****^p < 0.0001, ^***^p < 0.001, ^**^p < 0.01, ^*^p < 0.05. (**B**) To further understand the functional implications of UBR1 expression, a pan-cancer GSEA was conducted. This analysis identified significant enrichment of UBR1 in various signaling pathways across different cancer types.

Pan-cancer analysis was conducted using TCGA in conjunction with the GTEx database to determine UBR1 expression levels in normal and tumor tissues due to the limited number of normal tissue samples in TCGA. This analysis revealed that UBR1 is expressed in various types of cancer, including breast invasive carcinoma (BRCA), cervical ductal adenocarcinoma (CESC), cholangiocarcinoma (CHOL), colonic adenocarcinoma (COAD), diffuse large B-cell lymphoma (DLBC), esophageal carcinoma (ESCA), glioblastoma multiforme (GBM), renal clear cell carcinoma (KIRC), renal cell carcinoma (KIRP), acute myeloid leukemia (LAML), low-grade glioma (LGG), hepatocellular carcinoma (LIHC), lung adenocarcinoma (LUAD), lung squamous cell carcinoma (LUSC), ovarian serous cystadenocarcinoma (OV), pancreatic adenocarcinoma (PAAD), prostate adenocarcinoma (PRAD), rectal adenocarcinoma (READ), cutaneous melanoma (SKCM), gastric cancer (STAD), testicular germ cell tumor (TGCT), thyroid cancer (THCA), thymoma (THYM), endometrial cancer (UCEC), and uterine carcinosarcoma (UCS) (*P* < 0.0001). Additionally, UBR1 was elevated in adrenocortical carcinoma (ACC), bladder urothelial carcinoma (BLCA), and kidney chromophobe (KICH) (*P* < 0.05). In total, 28 cancers showed elevated expression of this protein (Figure 1A). Subsequently, we conducted a pan-cancer GSEA to identify the potential pathways influenced by UBR1. The results indicated that UBR1 protein is associated with various signaling pathways across different cancers. Specifically, it was enriched in gastric adenocarcinoma (STAD) and linked to the NF-κB signaling pathway, mTOR signaling pathway, oxidative phosphorylation, mitosis, and DNA damage checkpoints (Figure 1B). These findings suggest that UBR1 is upregulated in most cancers, including STAD, and is associated with multiple signaling pathways and functions.

### Elevated expression and diagnostic potential of UBR1 in gastric cancer

**Figure 2:**
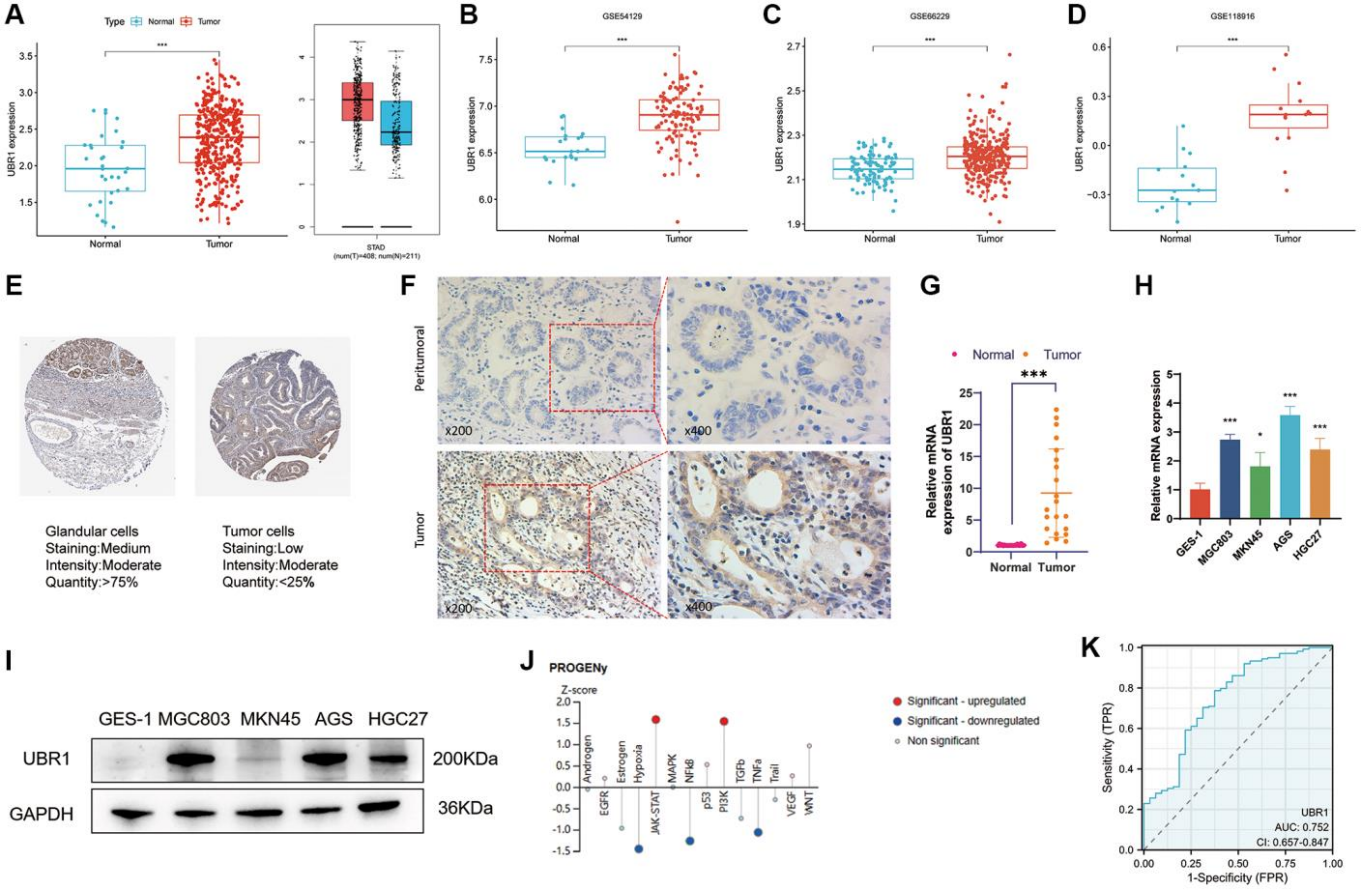
**Elevated expression and diagnostic potential of UBR1 in gastric cancer.** (**A**) UBR1 expression levels were analyzed using the TCGA and GTEx databases to provide a comprehensive overview of its expression in gastric cancer (GC). (**B**–**D**) To further validate and compare findings, UBR1 expression was examined across three GEO datasets: GSE54129, GSE66229, and GSE118916. (**E**) Immunohistochemical tissue microarray images from the HPA database highlighted UBR1 protein expression in various tissues. (**F**) Immunohistochemical protein expression levels of UBR1 in gastric cancer tissues and adjacent non-cancerous tissues. (**G**) Analysis of UBR1 mRNA levels in 21 pairs of GC and adjacent tissue samples showed significant differences, emphasizing its elevated expression in cancer tissues (^****^p < 0.0001, ^***^p < 0.001, ^**^p < 0.01, ^*^p < 0.05). (**H**) qPCR verification confirmed the elevated levels of UBR1 mRNA in four GC cell lines (MGC803, MKN45, AGS, and HGC27) compared to normal gastric mucosal cells (GES-1) (^****^p < 0.0001, ^***^p < 0.001, ^**^p < 0.01, ^*^p < 0.05). (**I**) Western blot analysis validated the protein expression of UBR1 in GES-1, MGC803, MKN45, AGS, and HGC27 cells, corroborating the mRNA findings. (**J**) Using data from the HPA database, predicted pathways and functions of UBR1 were identified, showing enrichment in AGS cells. (**K**) ROC curve analysis, based on TCGA data, evaluated the diagnostic potential of UBR1 in GC patients.

The association between UBR1 and GC development remains poorly understood. To analyze UBR1 expression in GC, we used the TCGA and GTEx databases (Figure 2A) and validated our findings with datasets from the GEO database, including GSE54129, GSE66229, and GSE118916 (Figure 2B–2D). UBR1 expression levels were significantly higher in GC tissues compared to normal tissues in both the TCGA and GEO datasets, consistent with results from the GEPIA database. Additionally, tissue microarray analysis from the HPA database using immunohistochemistry (IHC) showed significantly higher UBR1 levels in STAD tissues than in normal gastric tissues (Figure 2E). These experimental findings align with the bioinformatics analysis, indicating a significant increase in UBR1 expression in GC tissues compared to paired paraneoplastic tissues, as determined by IHC (Figure 2F).

We measured the mRNA expression of UBR1 in 21 paired carcinoma and paraneoplastic tissue samples and found that UBR1 mRNA levels were significantly elevated in GC tissues (*p* < 0.001) (Figure 2G). Furthermore, we analyzed UBR1 expression in gastric cancer cell lines. qPCR and WB detected the mRNA and protein levels of UBR1 in GES-1, MGC803, MKN45, AGS, and HGC27 gastric cancer cells, confirming significant overexpression in these cell lines compared to human gastric mucosal cells (GES-1) (Figure 2H, 2I). Next, we predicted potential pathways associated with UBR1 in AGS cells using the HPA database. Z-SCORE normalization demonstrated that UBR1 might be linked to various pathways in AGS cells, including NF-κB, JAK-STAT, PI3K, TNFα, and hypoxia (Figure 2J).

Finally, we evaluated the potential of UBR1 as a diagnostic biomarker for GC using ROC curve analysis. Based on the TCGA database, the ROC curve analysis revealed that UBR1 has potential as a diagnostic biomarker for patients, with an area under the curve (AUC) of 0.752 (Figure 2K). These findings suggest that UBR1 is overexpressed in GC tissues and cell lines compared to normal gastric mucosal tissues at the biochemical, transcriptional, and translational levels.

### High UBR1 expression is associated with poor prognosis in GC patients

**Figure 3:**
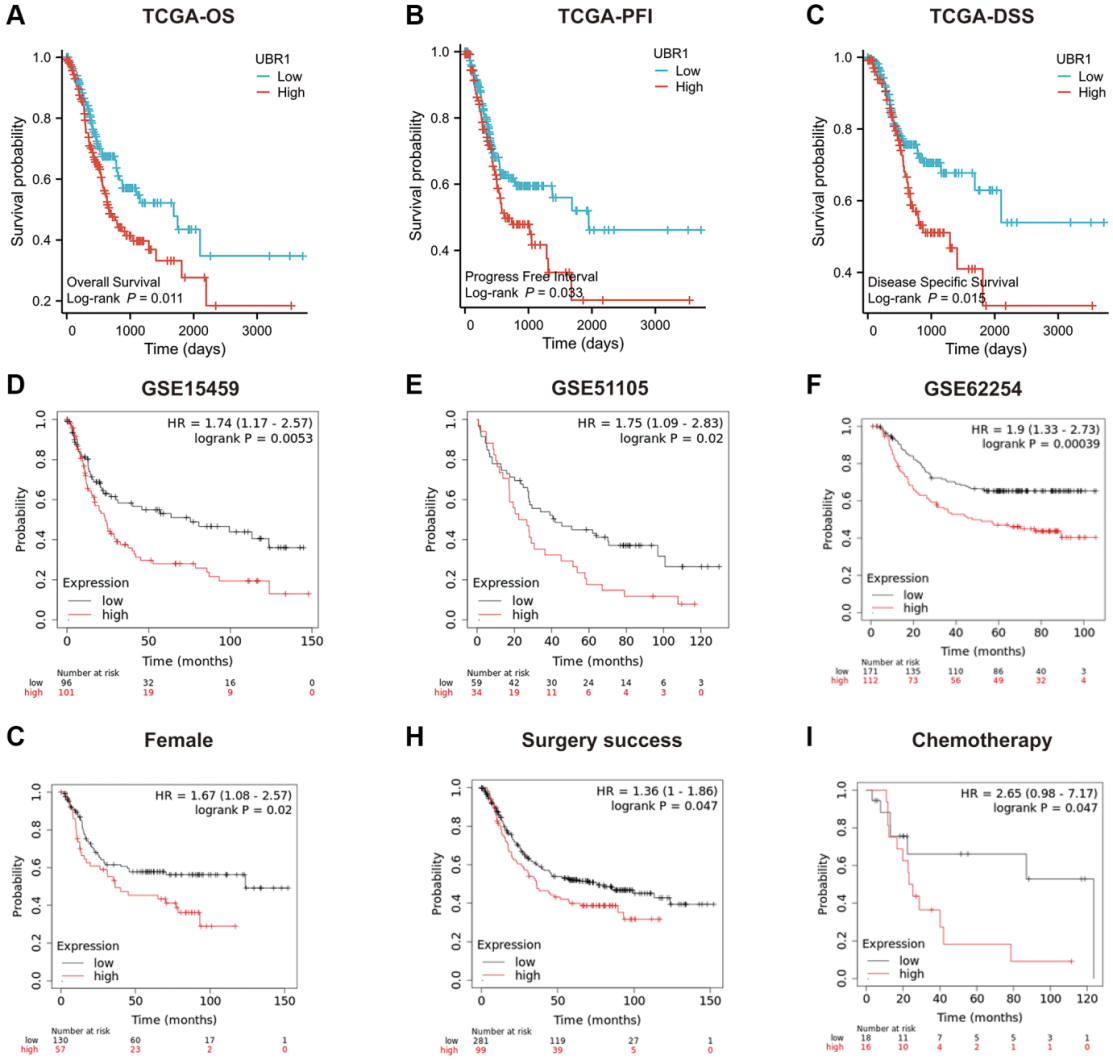
**Prognostic role of UBR1 in gastric cancer.** (**A**) Overall survival (OS) curves for patients expressing UBR1 were plotted using the Kaplan-Meier Plotter database, revealing a significant association (*p* < 0.05). (**B**) The progression-free interval survival curve also indicated a significant correlation with UBR1 expression (*p* < 0.05). (**C**) The disease-specific survival curve demonstrated a significant link (*p* < 0.05). Further validation was performed using OS data from GEO datasets:(**D**) GSE15459 (*p* < 0.01), (**E**) GSE51105 (*p* < 0.05), (**F**) GSE62254 (*p* < 0.0001). (**G**) subgroup analyses revealed significant OS differences based on UBR1 expression in: Female patients (*p* < 0.05). (**H**) Patients with GC who underwent successful surgery (*p* < 0.05). (**I**) GC patients treated with chemotherapy (*p* < 0.05).

We assessed the prognostic significance of UBR1 expression in patients with GC by analyzing TCGA and GEO data. The KM curves indicated a significant negative correlation between UBR1 expression and overall survival (OS), progression-free interval (PFI), and disease-specific survival (DSS) in patients (Figure 3A–3C). Additionally, high UBR1 expression in GEO datasets (GSE15459, GSE51105, and GSE62254) was positively correlated with poor prognosis of GC patients (Figure 3D–3F). Furthermore, gender was correlated with UBR1 expression, with higher expression of UBR1 associated with lower survival rates in women (Figure 3G). Patients with GC who had higher expression of UBR1 showed lower OS rates after surgery (Figure 3H) and a poorer response to chemotherapy (Figure 3I). These data suggest that UBR1 expression is associated with OS and plays a role in GC progression.

### Genetic alterations of UBR1 in gastric cancer

**Figure 4:**
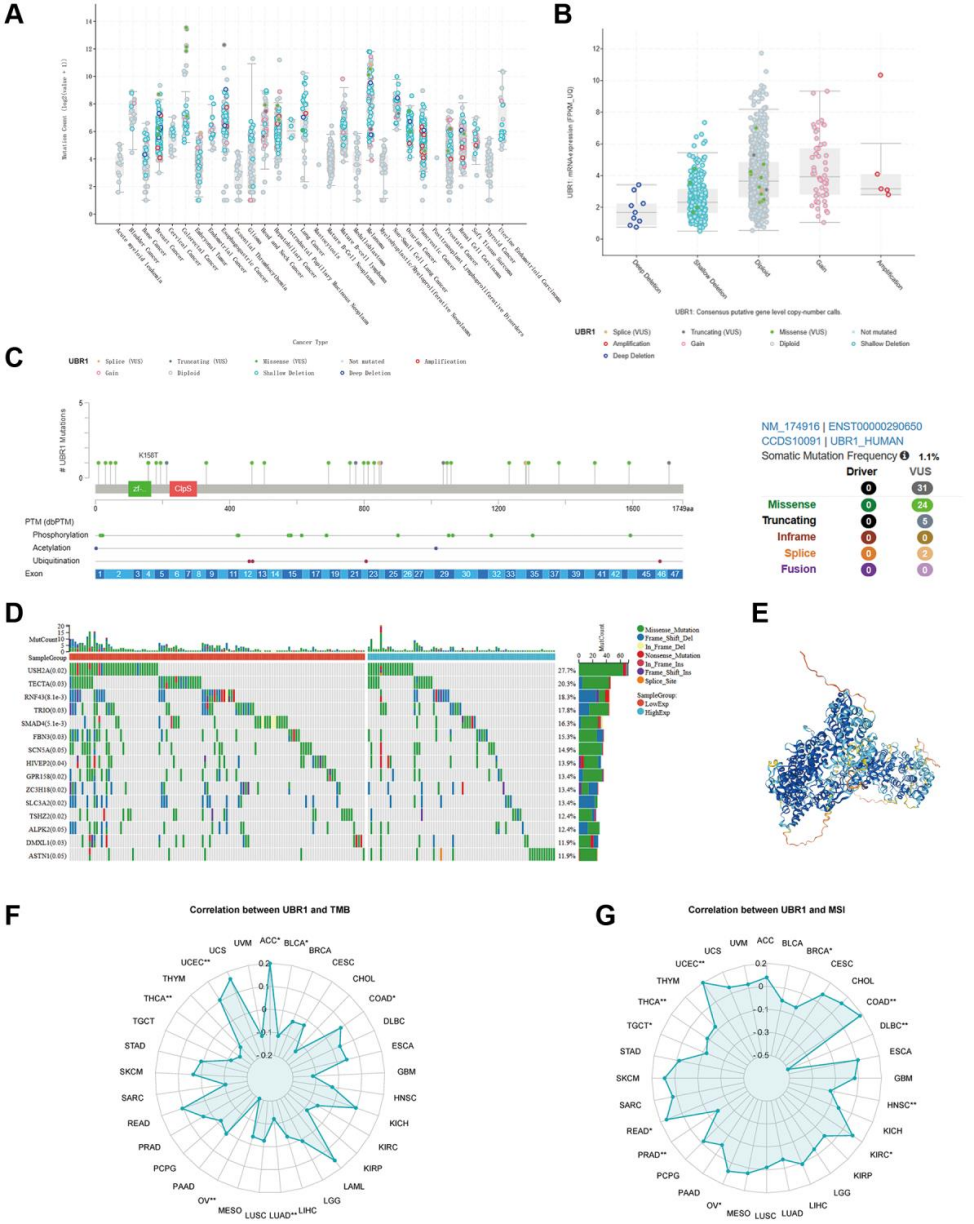
**Genetic alterations of UBR1 in gastric cancer.** (**A**) Analysis of the frequency of UBR1 mutations across various cancers using the cBioPortal database. (**B**) Frequency and types of genetic alterations of UBR1 in gastric cancer, including amplifications, deletions, and mutations. (**C**) Identification and analysis of UBR1 mutation sites, mutated amino acids, and their association with post-translational modifications, along with the overall frequency of these somatic mutations. (**D**) Mutation landscape of the top 15 genes with the highest frequency of UBR1 mutations in gastric cancer. (**E**) 4D structure of the UBR1 protein retrieved from the HPA database. (**F**, **G**) Correlation of UBR1 with Tumor Mutation Burden (TMB) and Microsatellite Instability (MSI) in malignancies, presented in radar charts.

We investigated the genetic modifications of UBR1 in human malignancies due to its anomalous expression in cancer. Using cBioPortal database analysis, we predicted the frequency of UBR1 mutations across various cancers (Figure 4A). In gastric cancer, UBR1 exhibited frequent genetic alterations including amplifications, profound deletions, and mutations, with a prevalence of 7.163% in three case studies (Figure 4B). We then identified the sites of UBR1 mutations, analyzed the mutated amino acids, and examined their association with post-translational modifications, as well as the overall frequency of these somatic mutations (Figure 4C). Subsequently, we constructed a mutation landscape for the top 15 genes with the highest frequency of UBR1 mutations in gastric cancer (Figure 4D). The 4D structure of the UBR1 protein was retrieved from the HPA database (Figure 4E). Finally, we explored the correlation of UBR1 with Tumor Mutation Burden (TMB) and Microsatellite Instability (MSI) in malignancies, presenting our findings in radar charts (Figure 4F, 4G).

### UBR1 expression is associated with GC progression

**Figure 5:**
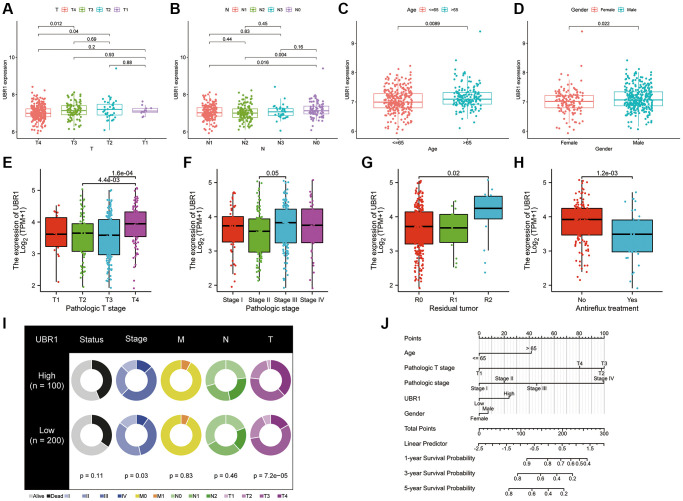
**UBR1 expression is associated with GC progression.** (**A**) UBR1 expression levels in patients with stage T3 and T4 gastric cancer (GC) compared to those with stage T2, based on the GEO dataset GSE84437. (**B**) UBR1 expression levels in patients with stage N1 and N2 GC compared to those with stage N0, from the GEO dataset GSE84437. (**C**) UBR1 expression levels in GC patients aged 65 years and older compared to younger patients, using data from the GEO dataset GSE84437. (**D**) UBR1 expression levels in male GC patients compared to female patients, based on the GEO dataset GSE84437. (**E**) UBR1 expression levels in patients with T4 stage GC, according to TCGA database data. (**F**) UBR1 expression levels in stage III GC patients compared to stage II patients, from the TCGA database. (**G**) UBR1 expression levels in tumors with sarcoid residual (R2) compared to those with complete tumor resection (R0), based on TCGA data. (**H**) UBR1 expression levels in patients who did not receive anti-reflux treatment, according to TCGA data. (**I**) Fan charts illustrating variations in UBR1 expression across different clinical prognostic stages. (**J**) Nomograms showing the relationship between UBR1 expression and various clinical prognostic factors in GC.

This study evaluated the involvement of UBR1 in gastric cancer (GC) by examining the correlation between UBR1 expression and various clinical parameters, including age, gender, tumor grade, residual tumor grade, anti-reflux treatment, and TNM stage. Our analysis utilized data from the GEO dataset GSE84437 and the TCGA database. We observed that UBR1 was upregulated in patients with stage T3 and T4 GC compared to those with stage T2 GC, as evidenced by data from the GEO database (Figure 5A). Similarly, UBR1 expression was higher in patients with stage N1 and N2 GC than in those with stage N0 GC (Figure 5B). Additionally, UBR1 expression was significantly elevated in GC patients aged 65 years and older compared to younger patients (Figure 5C). Male GC patients exhibited significantly higher UBR1 expression compared to female patients (Figure 5D). In contrast, data from the TCGA database indicated an increase in UBR1 expression in patients with T4 stage GC (Figure 5E) and higher expression in stage III GC patients compared to stage II patients (Figure 5F). Furthermore, UBR1 was significantly upregulated in tumors with sarcoid residual (R2) compared to those with complete tumor resection (R0) (Figure 5G). UBR1 expression was also increased in patients who did not receive anti-reflux treatment (Figure 5H). Fan charts and nomograms were employed to illustrate the variation in UBR1 expression across different clinical prognostic stages (Figure 5I, 5J). These findings suggest that various clinical factors may influence UBR1 expression and its role in the progression of GC. Elevated levels of UBR1 could serve as an unfavorable prognostic factor for GC patients. The baseline information table for UBR1 in GC patients from the TCGA database is provided in Supplementary Table 1.

### Biological functions of UBR1

**Figure 6:**
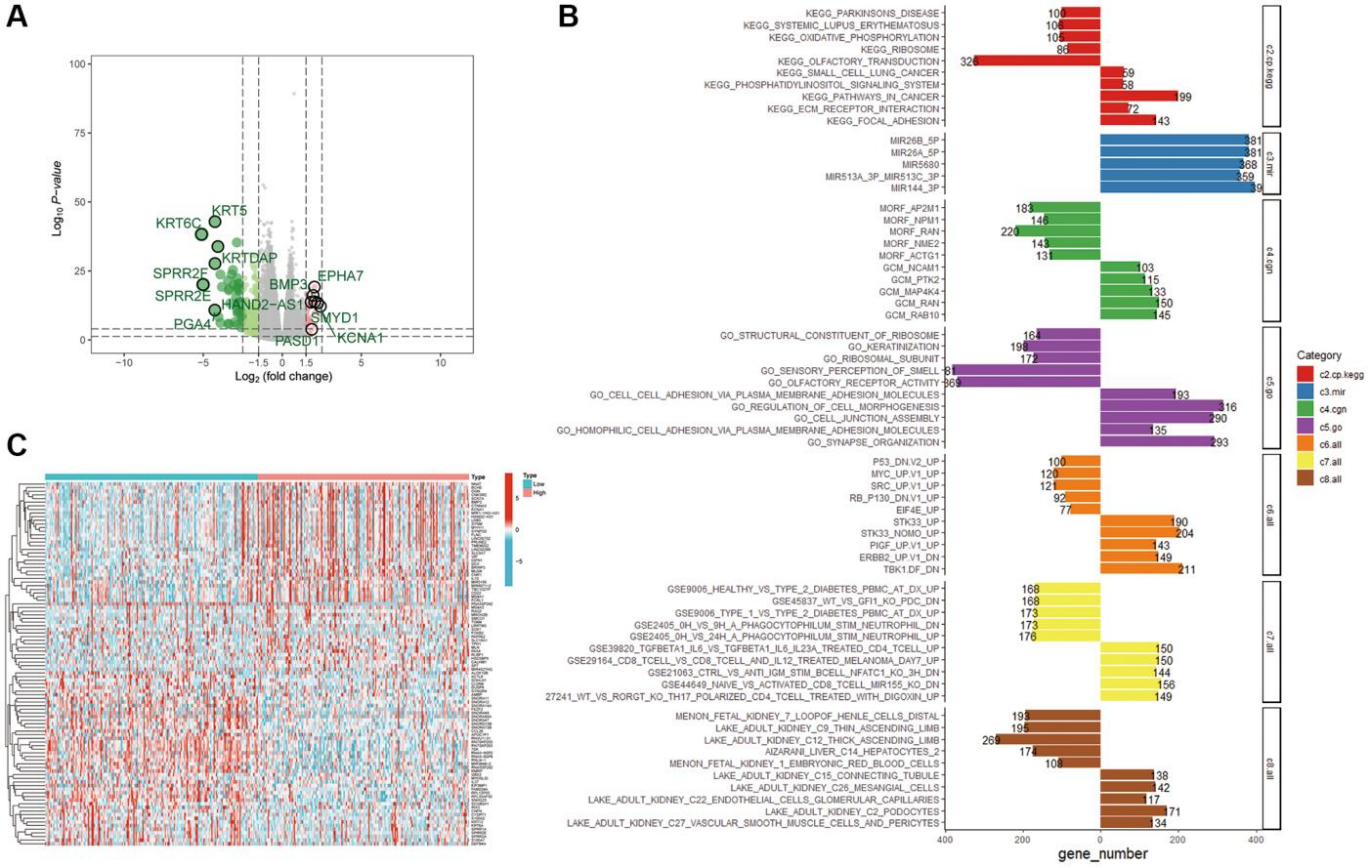
**Enrichment analysis of the UBR1 functional network.** (**A**) Volcano plots were utilized to examine the correlation between UBR1 and differentially expressed genes in gastric cancer (GC), with significance set at *p* < 0.05. (**B**) A heat map illustrating the differentially expressed genes associated with UBR1 in GC. (**C**) KEGG and GO pathway analyses were performed on UBR1-regulated genes, displaying the enrichment of downregulated genes on the left and upregulated genes on the right.

Multiple analyses were performed to determine the biological functions of UBR1 in gastric cancer (GC). We utilized the TCGA database to identify differentially expressed genes related to UBR1 in gastric cancer, as depicted in the volcano plot (Figure 6A). A heat map was then generated to further visualize the differential expression patterns of UBR1 (Figure 6B). We conducted separate KEGG and GO analyses for upregulated and downregulated genes (Figure 6C). KEGG analysis indicated that downregulated genes were enriched in pathways associated with Parkinson’s disease, systemic lupus erythematosus (SLE), oxidative phosphorylation, ribosome metabolism, and olfactory transmission. Conversely, upregulated genes were enriched in small cell lung cancer pathways, phospholipid acyl signaling, general cancer pathways, ECM receptor interactions, and adhesion processes. Additionally, we sought differentially expressed genes enriched among miRNA target genes (MIR). A comprehensive analysis of a vast array of computational genes defined by cancer-specific microarray data was performed. In the GO analysis, downregulated genes were mainly enriched in structural components of ribosomes, keratinization, ribosomal subunits, olfactory sensory perception, and olfactory receptor activity. Upregulated genes predominantly featured enhancements in cellular functions such as cell adhesion via plasma membrane adhesion molecules, regulation of cellular molecules, cell attachment components, homotypic cell adhesion, synaptic organization, and other cytological functions. Furthermore, we identified gene sets that represent disrupted cellular pathways in cancer, focusing particularly on downregulated genes such as P53, MYC, and SRC. Using the GEO database, we enriched both upregulated and downregulated gene sets associated with CD4+ and CD8+ immune cells to study cellular states and immune system perturbations. Finally, single-cell sequencing of human tissues was employed to identify cell-type markers and enriched gene sets for these clusters. Overall, our findings suggest that the differential gene sets for UBR1 are significantly involved in cancer pathways, P53 association, immune cell interactions, and multiple metabolic pathways.

### Analysis of the correlation between UBR1 expression and immunity

**Figure 7:**
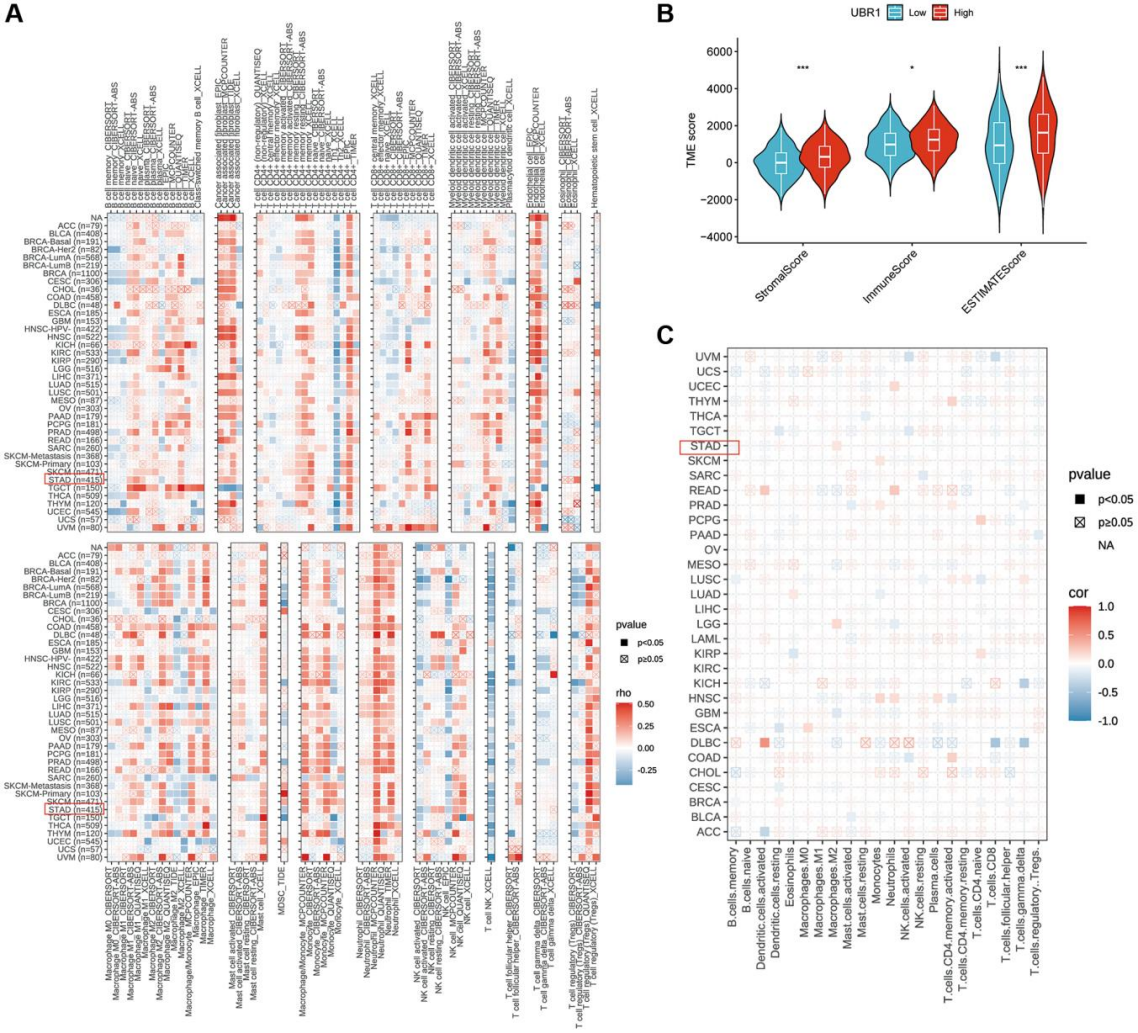
**Correlation between UBR1 expression and immune cell infiltration.** (**A**) The correlation between UBR1 expression and infiltrating immune cells was analyzed using the TIME algorithm. (**B**) Differences in immune, stromal, and ESTIMATE scores between high and low UBR1 expression groups were examined. (**C**) The EPIC algorithm was used to evaluate the expression of UBR1 and its relationship with immune-infiltrating cells. Significance levels are indicated as follows: ^*^P < 0.05; ^**^P < 0.01; ^***^P < 0.001; ^****^P < 0.0001; ns, not significant.

We utilized the TIMER and EPIC algorithms to assess the immune cell infiltration of UBR1 in individual tumors and investigated the correlation between UBR1 expression and immune infiltration levels, as shown in (Figure 7A, 7C). Subsequently, we compared the immune, stromal, and ESTIMATE scores between groups with high and low UBR1 expression. The results revealed significant differences in the immune and ESTIMATE scores (*p* > 0.001) (Figure 7B). The TIMER algorithm demonstrated a significant positive correlation between UBR1 expression and CD4+ T-cell infiltration in stomach adenocarcinoma (STAD) (*P* < 0.05). This association was also confirmed using the EPIC algorithm, which linked UBR1 expression with various cancers. Specifically, high UBR1 expression correlated positively with numerous cancers, including UVM, UCS, UCEC, THYM, THCA, TGCT, SKCM, PAAD, OV, MESO, LUAD, LIHC, LGG, LAML, KIRP, KIRC, HNSC, GBM, ESCA, COAD, CESC, BRCA, BLCA, and ACC. This correlation was further validated through the TIME algorithm.

### UBR1 interacts with PDL1 in GC

**Figure 8:**
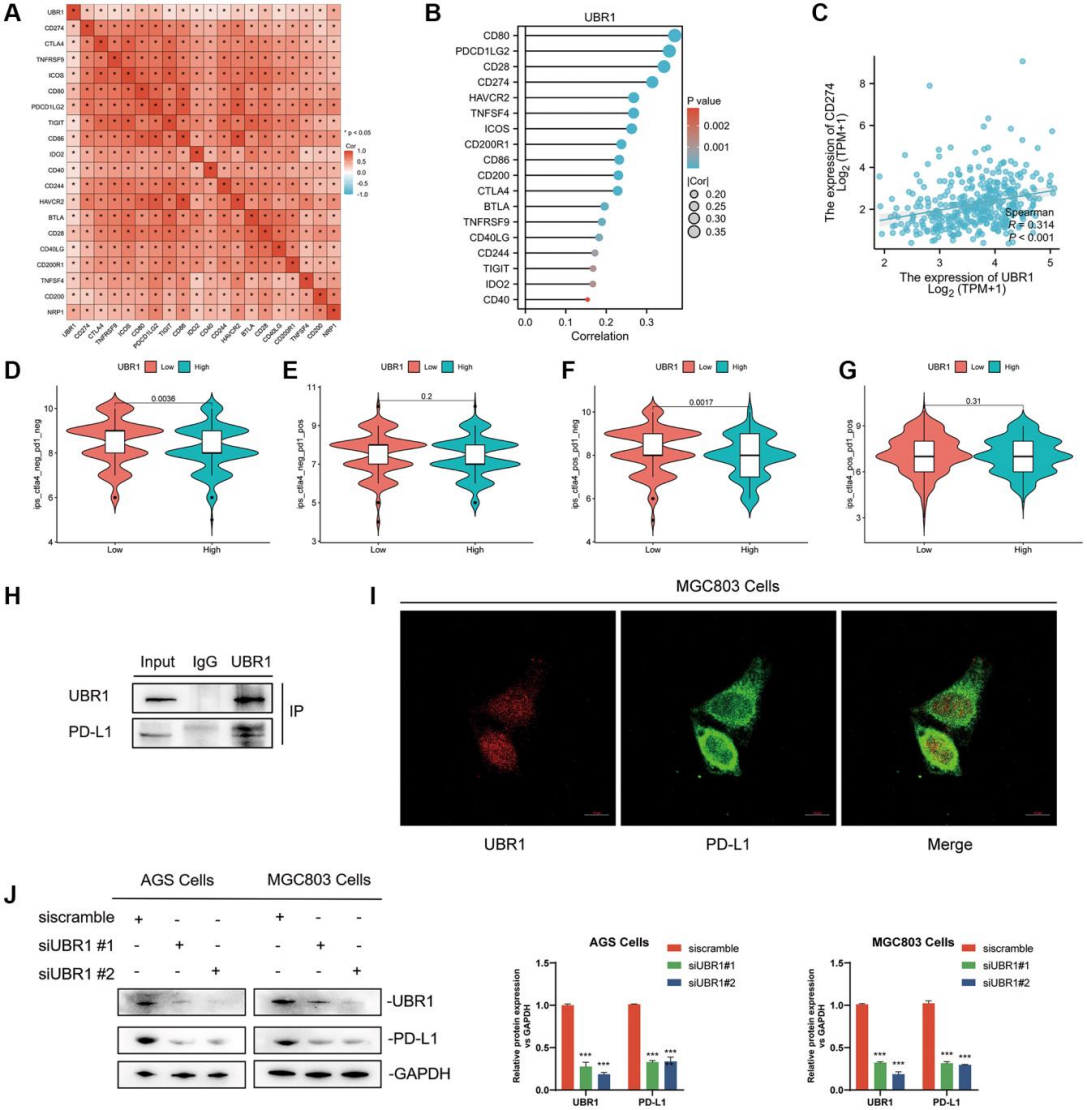
**Interaction between UBR1 and PD-L1 in Gastric Cancer.** (**A**) The results indicated a statistically significant correlation (*p* < 0.05) between UBR1 and various immune checkpoint-related genes, including PD-L1, CTLA4, TNFRS, ICOS, CD80, PDCD1LG2, TIGIT, CD86, IDO2, CD40, CD244, HAVCR2, BTLA, CD28, CD40LG, CD200R1, TNFSF4, CD200, and NRP1. (**B**) These genes were ranked by correlation size and presented in a lollipop plot, highlighting the top five genes: CD80, PDCD1LG2, CD28, PD-L1, and HAVCR2. (**C**) The correlation between UBR1 and PD-L1 in stomach adenocarcinoma (STAD) was further validated using the TCGA database, showing a significant positive correlation (R = 0.314, *p* < 0.001). (**D**–**G**) Immune scores and responses to immunotherapy were analyzed in UBR1 low- and high-expression groups, revealing significant differences in response among different patient cohorts. (**H**) Immunoprecipitation assays in MGC803 cells confirmed the binding affinity between UBR1 and PD-L1 proteins. (**I**) Immunofluorescence assays in MGC803 cells verified the colocalization of UBR1 and PD-L1 proteins. (**J**) Analysis of PD-L1 protein expression in null and UBR1 knockdown cell lines (AGS and MGC803) showed decreased PD-L1 expression following UBR1 knockdown.

This study further investigated the correlation between UBR1 and immune checkpoint genes by generating a heatmap. The results indicated that UBR1 had a statistically significant correlation (*p* < 0.05) with various immune checkpoint-related genes, including PD-L1, CTLA4, TNFRS, ICOS, CD80, PDCD1LG2, TIGIT, CD86, IDO2, CD40, CD244, HAVCR2, BTLA, CD28, CD40LG, CD200R1, TNFSF4, CD200, and NRP1 (Figure 8A). These genes were ranked based on the correlation size and presented in a lollipop plot, highlighting the top five genes: CD80, PDCD1LG2, CD28, PD-L1, and HAVCR2 (Figure 8B). The correlation between UBR1 and PD-L1 in stomach adenocarcinoma (STAD) was further validated using the TCGA database. The analysis showed a significant positive correlation between UBR1 and PD-L1 (R = 0.314, *p* < 0.001) (Figure 8C). In the context of immunotherapy, the UBR1 low-expression group exhibited higher immune scores among patients who were negative for both PD-1 and CTLA4, and this group also demonstrated a significantly better response to immunotherapy (*p* < 0.01) (Figure 8D). In patients positive for CTLA4, the UBR1 low-expression group showed higher immune scores compared to the UBR1 high-expression group, suggesting a better response to anti-CTLA4 treatment within the CTLA4 low-expression cohort (*p* < 0.01) (Figure 8F). However, in patients positive for both PD-1 and CTLA4, no statistically significant difference in immunotherapy response was observed between the high and low UBR1 expression groups (Figure 8E, 8G). To further elucidate the interaction between UBR1 and PD-L1, immunoprecipitation assays were performed in MGC803 cells, confirming that UBR1 and PD-L1 proteins exhibit binding affinity (Figure 8H). Additionally, immunofluorescence assays in MGC803 cells verified the colocalization of UBR1 and PD-L1, indicating spatial overlap between the two proteins (Figure 8I).

Finally, null and knockdown transformed cell lines were constructed using AGS and MGC803 cells, respectively. Following UBR1 knockdown, a decrease in PD-L1 protein expression was observed (Figure 8J). In conclusion, UBR1 is associated with immunotherapy and directly affects PD-L1 expression.

### UBR1 knockdown inhibits proliferation, migration and promotes apoptosis in GC cells *in vitro*

**Figure 9:**
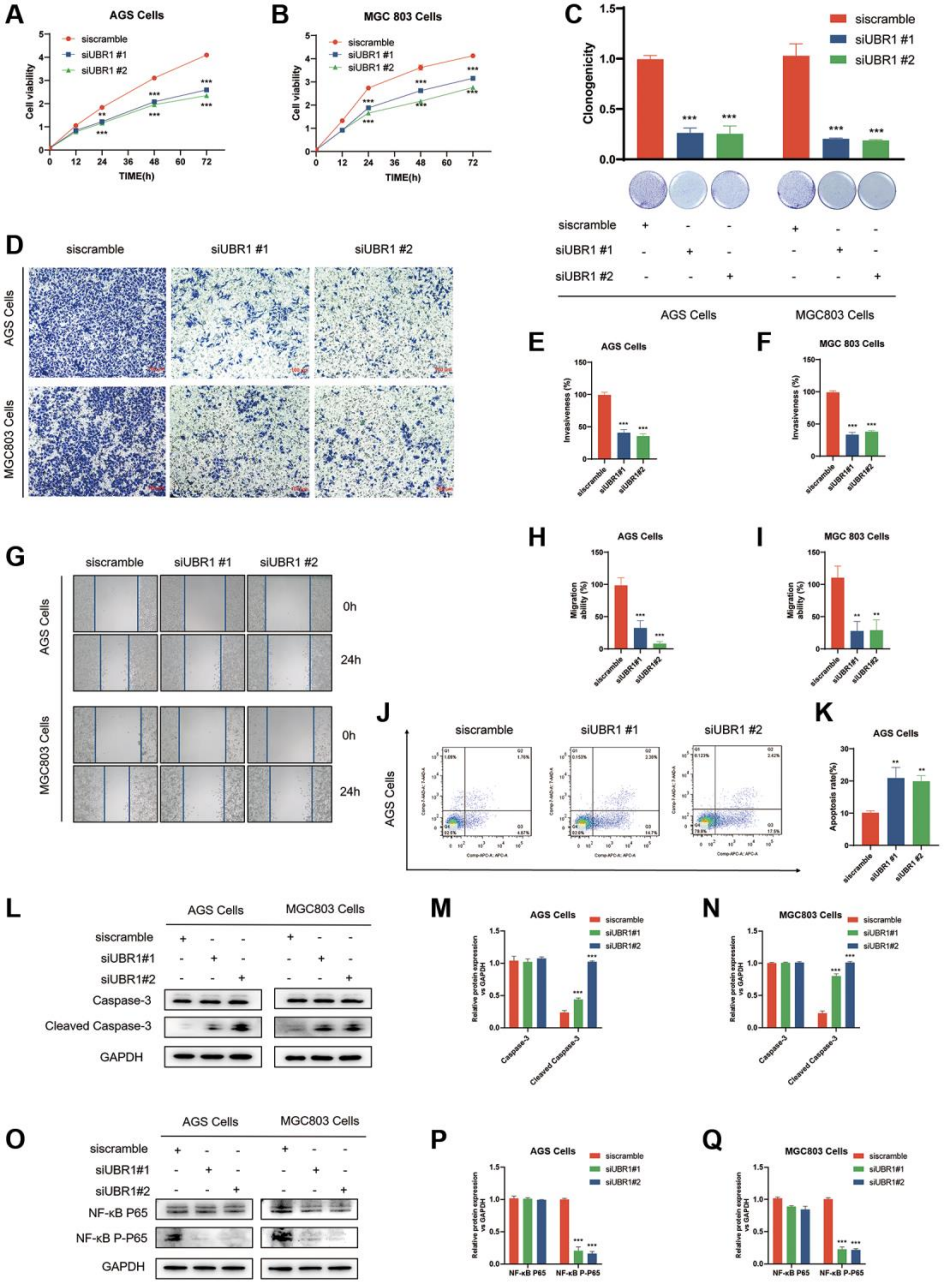
**Effects of UBR1 knockdown on proliferation, colony formation, invasion, migration, apoptosis, and NF-κB P65 pathway in AGS and MGC803 cells.** (**A**, **B**) Cell Counting Kit-8 (CCK8) assay results show that cell proliferation is significantly inhibited in AGS and MGC803 cells at 24, 48, and 72 hours post-UBR1 knockdown (*p* < 0.001). (**C**) Colony formation assay indicates a significant reduction in colony formation ability in both cell lines following UBR1 knockdown. (**D**–**F**) Transwell invasion assays demonstrate a significant decrease in the invasive capacity of AGS and MGC803 cells with UBR1 knockdown (*p* < 0.001). (**G**–**I**) Wound healing assays reveal a significant reduction in the migratory ability of UBR1-knockdown cells (*p* < 0.001). (**J**, **K**) Apoptosis analysis shows an increase in apoptosis in AGS cells post-UBR1 knockdown (*p* < 0.01). (**L**–**N**) Western blot analysis of apoptosis-related proteins Caspase-3 and Cleaved Caspase-3 indicates an increase in Cleaved Caspase-3 expression in both cell lines after UBR1 knockdown. (**O**–**Q**) Western blot analysis of NF-κB pathway proteins shows a significant reduction in phosphorylated NF-κB P65 expression in UBR1-knockdown cells, suggesting involvement in the NF-κB pathway. In all panels, data are presented as mean ± standard deviation (SD), and statistical significance is indicated with asterisks (^*^p < 0.05, ^**^p < 0.01, ^***^p < 0.001).

High gene expression is often associated with poor prognosis. To elucidate the function of UBR1 in gastric cancer (GC), we used two siRNAs for cis-translational knockdown in AGS and MGC803 cells. Prior to the experiment, we evaluated the knockdown efficiency using Western blot (WB) analysis. By knocking down UBR1 in AGS and MGC803 cells, we assessed the impact of UBR1 silencing on the proliferative capacity of GC cells. According to the Cell Counting Kit-8 (CCK8) assay, cell growth significantly decreased at 24, 48, and 72 hours (*p* < 0.001) (Figure 9A, 9B). Additionally, UBR1 knockdown in both cell lines resulted in a statistically significant reduction in colony formation (Figure 9C). In the Transwell invasion assays using Matrigel gel, UBR1 knockdown led to a significant decrease in the invasive ability of AGS and MGC803 cells (*p* < 0.001) (Figure 9D–9F). Furthermore, in the wound healing assay, UBR1 knockdown reduced migratory capacity (*p* < 0.001) (Figure 9G–9I) and induced apoptosis in AGS cells (*p* < 0.01) (Figure 9J, 9K). To confirm these findings, we verified the expression of apoptosis-related proteins Caspase-3 and Cleaved Caspase-3 using WB. Knocking down UBR1 induced an increase in Cleaved Caspase-3 protein expression in both cell types, as shown in bar charts (Figure 9L–9N).

Previous analyses, including GESA and HPA, predicted that UBR1 might be enriched in the NF-κB pathway. This prediction was validated through knockdown experiments in AGS and MGC803 cells. UBR1 knockdown resulted in a significant reduction in phosphorylated NF-κB P65 expression, as indicated by WB (Figure 9O–9Q).

**Figure 10:**
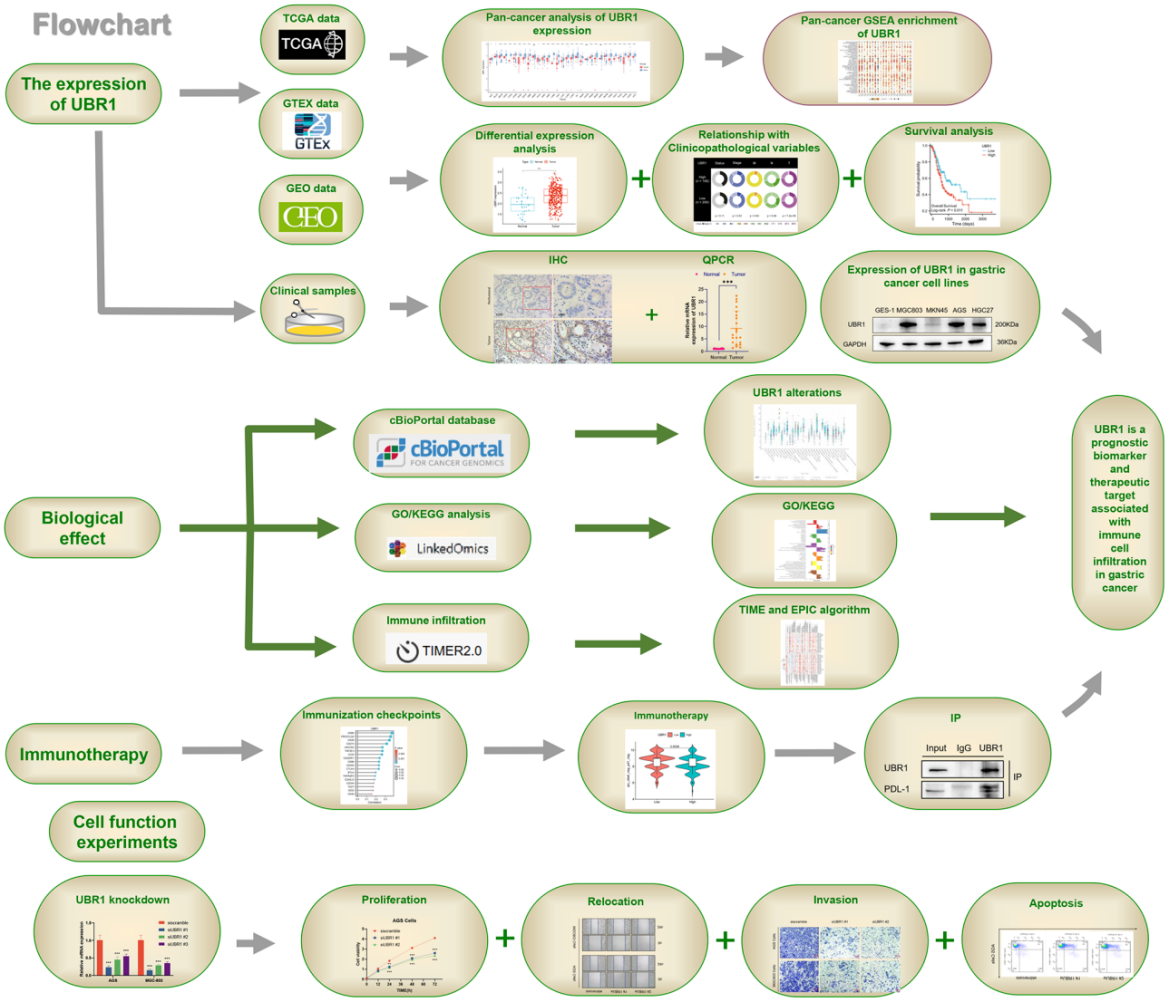
**Study workflow.** UBR1 is a prognostic biomarker and therapeutic target associated with immune cell infiltration in gastric cancer.

The study workflow detailed in this research elaborates on the role of UBR1 in gastric cancer and its potential clinical applications (Figure 10). In summary, UBR1 expression affects various cellular processes, including cell growth, colony formation, invasion, migration, and apoptosis. It is also enriched in the NF-κB P65 pathway in GC cell lines, highlighting its potential as a therapeutic target.

## DISCUSSION

GC is one of the most prevalent cancers, with poor prognosis, significant incidence, and mortality rates globally. Early detection of GC is challenging, resulting in many patients being diagnosed at an advanced stage [20], leading to poor prognosis and high recurrence rates despite surgical intervention. Therefore, identifying new therapeutic targets and biomarkers for the early diagnosis of GC has the potential to improve the prognosis of patients [21].

The 200 kDa E3 ligase UBR1 is a widely conserved post-translationally modified protein involved in almost all cellular processes [22]. Mutations in UBR1 result in Johnson-Blizzard syndrome, an autosomal recessive disorder characterized by pancreatic insufficiency, multiple malformations, and mental retardation [15]. A study published by the Zhao and Liu group in Nature elucidated the structural mechanism of the sequential catalytic process of ubiquitin ligases [12]. Despite recent progress in understanding its dynamic mechanism for recognizing ubiquitinated substrates, the functional and cellular pathway implications of UBR1 in tumors are not well understood. This study established the correlation between UBR1 expression and GC development through a combination of *in vitro* experiments and raw data analysis. We also investigated the effect of UBR1 expression on PDL1 and its potential as a biomarker for immunotherapy. First, UBR1 was found to be overexpressed in various types of cancers, particularly GC, which is consistent with previous studies. Previous research has also shown increased expression of cyclic UBR1 in breast [23] and lung [24] cancers. Our study using BioSign predicted that high expression of UBR1 in GC correlates with unfavorable pathological features and poor prognosis. Aberrant mutations in UBR1, including nonsense, shift, splice sites, missense mutations, and small in-frame deletions [25], are linked to various diseases, which is largely consistent with our predictions. These findings suggest a potential correlation between UBR1 dysregulation (high or low expression, or loss of function) and GC onset and progression.

Tumor immunity is a crucial aspect of the tumor microenvironment. Although CD8+ T cells remain the primary focus of therapeutic target research, CD4+ T cells are increasingly being studied for their role in tumor immunity [26], However, the mechanisms underlying their activation are not yet fully understood. Identifying the molecules and immune markers responsible for CD4+ T-cell activation is a critical concern in tumor immunotherapy. In this study, BioSign predicted a significant correlation between UBR1 expression and immune cell infiltration in GC. First, the ESTIMATE software predicted higher stromal and immune scores for both high and low expression of UBR1 in the tumor microenvironment (TME). In pan-cancer analysis using the TIME and EPIC algorithms, UBR1 was found to be associated with various immune cells, particularly CD4+ T cells. Further analysis was conducted to compare patients with high and low UBR1 expression receiving CTLA-4 and PD-1 immunotherapy and to examine the correlation between UBR1 and PDL1. The results indicated a significant positive correlation between UBR1 and PDL1. Immunoprecipitation (IP) experiments were conducted to establish the direct effect of UBR1 on PDL1 in AGS cells. Knockdown of UBR1 resulted in a reduction in PDL1 at both the mRNA and protein levels.

Previous studies have extensively investigated the structure-function relationship of UBR1. However, limited research has explored its implications in cancer, and its effects on gastric cancer (GC) remain unclear. This study is the first to investigate the effects of UBR1 expression on GC cell proliferation, invasion, migration, and apoptosis. To assess these effects, siRNA was used to knockdown UBR1 protein expression, which was subsequently confirmed using qPCR and Western blot (WB) analyses. UBR1 knockdown inhibited proliferation, invasion, and migration, while also inducing apoptosis in AGS and MGC803 cells. These findings demonstrate that high UBR1 expression in gastric cancer may be a risk factor for poor prognosis.

However, this study has some limitations. First, our transcriptomic and clinical data were obtained from publicly available databases, which may have introduced some systematic bias. Second, we used qPCR to detect UBR1 expression in 21 paired cancer and paraneoplastic tissue samples. Additional samples and protein analyses are required to confirm UBR1 expression in GC. Third, we did not investigate the molecular mechanisms underlying UBR1’s role in immune cell infiltration or its effects on GC cell proliferation, invasion, migration, and apoptosis. Overall, this study identifies UBR1 as a potential biomarker for the diagnosis and prognosis of GC patients. Additionally, UBR1 appears to be a promising target for immunotherapy, warranting further investigation.

## CONCLUSIONS

The analysis of public data, databases, and our experimental results demonstrate that UBR1 can serve as a diagnostic and prognostic biomarker for GC. UBR1 can modulate PDL1 expression, which may have immunotherapeutic implications. Regulation of the NF-κB pathway by UBR1 warrants further validation through clinical, cytological, and animal experiments. This study provides a basis for UBR1 to explore potential therapeutic targets in GC.

## Supplementary Materials

Supplementary Table 1
