# Supplementary material for: UBR1 is a prognostic biomarker and therapeutic target associated with immune cell infiltration in gastric cancer
Source: Aging (Albany NY). 2024 Aug 23;16(16):12029–49. doi: 10.18632/aging.206079 (PMC11386912; doi:10.18632/aging.206079)
Supplement: Supplementary Table 1 [file aging-16-206079-s001.pdf]

SUPPLEMENTARY TABLE

Supplementary Table 1. Baseline information table for UBR1 in the TCGA database.

Characteristics	Low expression of UBR1	High expression of UBR1	*P*-value
n	187	188	
Pathologic T stage, *n* (%)			<0.001
T1 and T2	57 (15.5%)	42 (11.4%)	
T3	96 (26.2%)	72 (19.6%)	
T4	34 (9.3%)	66 (18%)	
Pathologic N stage, *n* (%)			0.869
N0	59 (16.5%)	52 (14.6%)	
N1	48 (13.4%)	49 (13.7%)	
N2	39 (10.9%)	36 (10.1%)	
N3	35 (9.8%)	39 (10.9%)	
Pathologic M stage, *n* (%)			0.893
M0	167 (47%)	163 (45.9%)	
M1	13 (3.7%)	12 (3.4%)	
Pathologic stage, *n* (%)			0.291
Stage I	28 (8%)	25 (7.1%)	
Stage II	64 (18.2%)	47 (13.4%)	
Stage III	69 (19.6%)	81 (23%)	
Stage IV	18 (5.1%)	20 (5.7%)	
Primary therapy outcome, *n* (%)			0.726
PD	34 (10.7%)	31 (9.8%)	
SD	9 (2.8%)	8 (2.5%)	
PR	1 (0.3%)	3 (0.9%)	
CR	124 (39.1%)	107 (33.8%)	
Gender, *n* (%)			0.048
Female	76 (20.3%)	58 (15.5%)	
Male	111 (29.6%)	130 (34.7%)	
Age, *n* (%)			0.041
≤65	92 (24.8%)	72 (19.4%)	
>65	94 (25.3%)	113 (30.5%)	
Histological type, *n* (%)			0.648
Diffuse type	33 (8.8%)	30 (8%)	
Mucinous type	11 (2.9%)	8 (2.1%)	
Not otherwise specified	101 (27%)	106 (28.3%)	
Papillary type	3 (0.8%)	2 (0.5%)	
Tubular type	36 (9.6%)	33 (8.8%)	
Signet ring type	3 (0.8%)	8 (2.1%)	
Antireflux treatment, *n* (%)			<0.001
No	51 (28.5%)	91 (50.8%)	
Yes	25 (14%)	12 (6.7%)	
